# Music-space associations are grounded, embodied and situated: examination of cello experts and non-musicians in a standard tone discrimination task

**DOI:** 10.1007/s00426-017-0898-y

**Published:** 2017-07-25

**Authors:** Martin Lachmair, Ulrike Cress, Tim Fissler, Simone Kurek, Jan Leininger, Hans-Christoph Nuerk

**Affiliations:** 1 Department of Psychology, University of Tübingen, Tübingen, Germany; 2 Leibniz-Institut für Wissensmedien, Schleichstraße 6, 72076 Tübingen, Germany; 3 LEAD Graduate School and Research Network, Tübingen, Germany

## Abstract

In recent research, a systematic association of musical pitch with space has been described in the so-called Spatial-Pitch-Association-of-Response Codes-effect (SPARC). Typically, high pitch is associated with upper/right and low pitch with lower/left space. However, a theoretical classification of these associations regarding their experiential sources is difficult. Therefore, we applied a theoretical framework of numerical cognition classifying similar Space-Associated Response Codes (SARC) effects according to their groundedness, embodiedness and situatedness. We tested these attributes with a group of non-musicians and with a group of highly skilled cello players playing high tones with lower hand positions (i.e., reverse SPARC alignment) in a standard SPARC context of a piano and a reversed SPARC context of a cello. The results showed that SPARC is grounded, in general. However, for cello player SPARC is also situated and embodied. We conclude that groundedness, embodiedness and situatedness provide general characteristics of mapping cognitive representations to space.

## Introduction

Over the last years there has been accumulating evidence that sensorimotor representations are at the core of information processing. Experiences stemming from interacting with the environment shape the way we represent objects, states or events mentally (Barsalou, 2010; Areshenkoff, Bub, & Masson, 2017; Lachmair, Ruiz Fernandez, Gerjets, Bury, Fischer, & Bock, 2016). Given that human cognition has evolved along with the human body, which operates within the constraints imposed by physical laws, it is unsurprising that cognition is strongly related to space. And indeed, findings from different cognitive domains provide evidence for the mapping of mental representations along spatial dimensions (cf. Lakoff & Johnson, 2008). These mappings are referred to as Space-Associated Response-Codes (SARC; e.g., Fitts & Seeger, 1953). SARC effects have been found in all three spatial dimensions, i.e., horizontally, vertically or sagittally, for example for linguistic stimuli (e.g., Lachmair, Duschig, De Fillippis, de la Vega, & Kaup, 2011; de la Vega, Dudschig, De Filippis, Lachmair, & Kaup, 2013; Lavender & Hommel, 2007), in numerical cognition (e.g., Dehaene, Bossini, & Giraux, 1993; Nuerk, Wood, & Willmes, 2005; for a review: Winter, Matlock, Shaki, & Fischer, 2015) and also in musical cognition (e.g., Rusconi, Kwan, Giordano, Umilta, & Butterworth, 2006; Drost, Rieger, Brass, Gunter, & Prinz, 2005; Stewart, Verdonschot, Kajihara, & Sparks, 2013b).

However, although the underlying mechanism of SARCs is theoretically well described (e.g., Hommel, 2015), it is often unclear what role the different characteristics of the spatial dimensions of SARCs play. The relevance of this issue will be demonstrated in the following study by focusing on a special SARC in more detail, namely the so-called Space Pitch Associated Response Code (SPARC). Recent literature in this field has reported spatial mappings on all spatial axes. For example, Rusconi et al. (2006) reported that the mental representation of pitch is mainly arranged along a vertical spatial axis independent of musical expertise. In their study, musicians and non-musicians performed a timbre judgment task of isolated tones. Both groups showed a pitch-effect in vertical space, showing faster upward responses and slower downward responses following high pitches, and the reverse pattern for low pitches. The results also revealed a horizontal mapping of pitch, but only for the musically trained group, showing faster responses to the right and slower responses to the left following high pitches, and the reverse pattern for low pitches. From this study can be concluded that the spatial associations of pitch originate from different experiential sources; the vertical association seems derived from more general experiences shaped by physical laws, affecting musicians and non-musicians in the same way, whereas the horizontal effect draws from experiences stemming from exercise with the piano.

Several studies have investigated the horizontal alignment of pitch systematically with musicians and non-musicians regarding perception-motor coupling (e.g., Repp & Knoblich, 2007, 2009; Taylor & Witt, 2015; Drost et al., 2005). For example, Stewart, Verdonschot, Nasralla and Lanipekun (2013a) investigated pitch effects for a horizontal alignment like that of a piano keyboard, but also for a reversed alignment, similar to a violin (Stewart et al., 2013b). One finding of these studies is that musicians compared to non-musicians show a clear pre-attentive auditory-motor coupling; a group of violinists as well as a group of pianists were affected by the pitch at which an aurally presented sequence of tones was presented. Sequences presented at pitches that were congruent with respect to the cued motor responses (i.e., for pianists a horizontal alignment from low tones to the left to high tones to the right; for violinists a sagittal alignment from low tones away and high tones towards the body) elicited faster responses compared to sequences where pitches and motor responses were incongruent (Stewart et al., 2013a, b). Crucially, the violin study also showed that for non-musicians no significant congruent/incongruent differences occurred in contrast to the piano study. The authors attribute this outcome to the employed paradigm: participants held a violin in the typical manner of a violinist, which does not involve spatial compatibility effects such as the SPARC effect. This may have contributed to the observed differences in the piano study (Stewart et al., 2013b).

A similar finding was reported in an earlier study by Drost, Rieger and Prinz (2007). They showed that responses of pianists and guitarists to auditory distractors in varying instrumental timbre were affected by the congruency between the timbre of the distractors and the instrumental expertise of the participants. Concretely, while presenting task-irrelevant auditory distractors, participants responded to visual stimuli by playing chords on their instrument. The auditory distractors were presented in one of five different timbres. The results showed an interference effect for pianists and guitarists with timbres of their own instrument, i.e., for pianists with piano-timbre and for guitarists with guitar timbre. The authors conclude that integrated action-effect associations primarily seem to consist of a specific component on a sensory-motor level involving the familiar instrument (Drost et al., 2007).

Hence, one can summarize that effects of pitch obviously depend on different spatially associated experiential representations that affect musicians and non-musicians in different ways. The sources for vertical and horizontal SPARC for example as described in Rusconi et al. (2006) for musicians and non-musicians are different: The vertical SPARC has its origin in a more fundamental source, namely physical laws like gravity, affecting musicians and non-musicians in the same way. However, the horizontal SPARC can be explained by the particular expertise of musicians and is additionally affected by the instrumental context. Thus, the question arises if the different origins also confer differences on the related SPARCs, for example, with regard to relative strength or robustness resulting from exercise.

### Embodied influences in Spatial Associations of Response Codes: the case of numbers

A similar flexibility with regard to spatial representations has also been observed in other cognitive domains. For example, findings show similar SARCs when processing numbers. The standard version of this Spatial Number Associated Response Codes-effect (SNARC-effect) describes that (in Western cultures) smaller numbers are responded to faster with the left response code and larger numbers with the right response code (Dehaene et al., 1993, for a meta-analysis and review see Fischer & Shaki, 2014). However, results of several studies suggest a vertical representation of numbers from bottom (small numbers) to top (larger numbers) as well. For instance, moving the head by looking upwards to the right produced more high numbers compared to low numbers and vice versa when looking downwards or to the left in a number generation task (Winter & Matlock, 2013). Moreover, the vertical SNARC-effect seemed to be more robust than the horizontal SNARC-effect, although a correlation between the vertical and the horizontal effects occurred. This might suggest a general sense for magnitude having its basis in physical experiences defined by physical conditions like gravity (e.g., Fischer, 2008; Fischer, 2012; cf. Walsh, 2003). However, if this is true, a theoretical framework is needed that can account for the flexible alignments of SNARC-effects.

Fischer and Brugger (2011; see also Wasner, Moeller, Fischer, & Nuerk, 2014) proposed such a framework: They suggested that spatial associations of numbers depend on grounded, embodied and situated aspects. However, due to the diverse use of the terms “grounded”, “embodied” and “situated” in literature (cf. Barsalou, 2010) some terminological clarifications were necessary, specific for their framework. Accordingly, groundedness assumes that human cognition evolved along with the human body, within strict limitations independent of culture. These limitations are caused by universal physical laws, such as earth’s gravity, which is a stable and ubiquitous reference for the vertical space. Along this line, a vertical SNARC-effect would be considered grounded because it would occur across cultures in every place on earth; just like the universal experience that pouring water in a glass would increase the water level. Accordingly, one can also assume that this effect would be rather robust (cf. Winter & Matlock, 2013) and hard to retrain because of its generality.

Second, embodiedness is based on life-long learned behavior due to idiosyncratic options of the human body when interacting with the world. Thus, embodiedness is closest to individual differences. An example for embodiedness was reported in a recent study by Huber et al. (2015). There, right- and left-handed participants conducted a parity judgment task, in which they had to decide whether a presented number was odd or even by pressing a left or right located response key. Typically, responses to even digits are faster to the right with the right hand and slower to the left with the left hand. The reverse pattern holds for odd digits. This so-called Markedness-of-Response Code (MARC) effect is explained with the linguistic markedness according to the correspondence of “even” and “right” which are supposed to be linguistically unmarked whereas “odd” and “left” are linguistically marked. Interestingly, the linguistic markedness account would not predict that handedness can moderate this MARC effect, because it should not affect the markedness of the words “left”, “right”, “odd”, and “even”. Nevertheless, the results of this study showed that handedness indeed modulated the MARC effect for the numbers. Whereas right-handers showed a regular MARC effect, this effect disappeared for left-handers. Moreover, a closer inspection revealed that the MARC effect in left-handers depended on the degree of left-handedness with a reversed MARC effect for most left-handed participants. The authors argue that this result reflects an embodied MARC effect in line with the body specificity hypothesis (Casasanto, 2009). Accordingly, “even” is a good and positive concept which is strongly associated with the dominant side of humans through life-long learned experiences according to bodily predisposition. In contrast, “odd” is a negative concept which is associated with the non-dominant side (Huber et al., 2015).

But embodiedness is also based on life-long learned behavior due to cultural constraints. For example, in Western cultures with reading direction from left to right, a SNARC-effect occurs associating low numbers with the left and high numbers with the right. Interestingly, it has been shown that this alignment is reversed, for example, in native Hebrew speakers, who read from right to left (Shaki & Fischer, 2012). Thus, following Myachykov and colleagues, such embodied representations can be considered as a core of offline representations, the parameters of which were shaped by the individual constraints of one’s body (Myachykov, Sheepers, Fischer, & Kessler, 2014).

Third, situatedness refers to the direct experience a specific context can elicit, which in turn can affect the cognition of an individual as well. For example, it has been shown that a reversed horizontal SNARC-effect (i.e., locating small numbers to the right and high numbers to the left) occurred when participants had to imagine a clock face (Bächtold, Baumüller, & Brugger, 1998). Interestingly, despite the deep experience from regularly looking at the clock, the specific alignment of numbers on a clock face does not generalize to horizontal space, presumably due to nonspecific embodied components. However, this deep experience seems to make individuals more vulnerable to the specific situation.

According to these examples, it can be concluded that the different experiential sources of groundedness, embodiedness and situatedness play obviously different roles for SNARC. Thus, the proposed framework of Fischer and Brugger provides a comprehensive hierarchical structure to account for the flexible alignments of SNARC. Moreover, a crucial claim of this framework is that the mapping flexibility of SNARC diminishes gradually from groundedness, where mapping is the most robust and hardest to retrain, through embodiedness to situatedness, which constitutes the most flexible mapping of SNARC (cf., Fischer & Brugger, 2011; Fischer 2012; Myachykov et al., 2014). Henceforth, we refer to the groundedness–embodiedness–situatedness framework as the GES-framework.

### Applying the GES-framework on SPARC

The GES-framework will now be applied to the SPARC effect. Accordingly, the mapping of pitch onto the vertical dimension of space with low pitches to the bottom and high pitches to the top is considered grounded. This is supported by the results showing a vertical arrangement of pitch independent of musical expertise and the ability to read and/or write musical notes (Roffl & Butler, 2005). In addition to that, Parise, Knorre and Ernst (2014) showed that natural sounds from the environment that come from high elevations are more likely to be higher in pitch. Thus, vertical space seems to reflect a universal reference frame of pitch height. This reference frame seems to be activated very automatically in human musical cognition (cf. Rusconi et al., 2006). Additionally, musical expertise affects SPARC as well (see above). It seems that playing the piano establishes a spatial reference frame along with the sensorimotor experiences with that instrument (e.g., Drost et al., 2005; Repp & Knoblich, 2007, 2009; Taylor & Witt, 2015). In terms of GES this would be considered embodiment, due to intensive cultural learning experiences. Here, however, we do not talk about cultural factors that may separate nations (like script or writing directions), but of factors that may separate sub-cultures within one nation, i.e., people heavily involved in musical culture vs. people who are not. And finally, it has been shown that the action-based and perceptual context related to musical expertise does influence SPARC (e.g., Drost et al., 2007). Again, it is important to note the difference between embodied and situated influences: embodied influences would not refer to the actual situation per se (e.g., the instrumental context), but to long-lasting learning experiences, which possibly build up certain directional representations beyond a specific situation.

Against this theoretical background, the question arises to which extent SPARC effects are grounded, embodied or situated in nature. The objective of the present study was to work that out. Therefore, we based the experimental setup of this study upon the paradigm which was used in Rusconi et al. (2006), whose results showed a vertical SPARC. Thus, a group of non-musicians and a group of cello players performed a tone discrimination task. The cello is an interesting instrument with regard to GES. On a cello, high or low pitches are generated by grasping the strings downwards towards the tailpiece, or upwards towards the scroll of the cello, respectively. This yields a spatial arrangement of pitches that is vertically inverted compared to the arrangement of standard SPARC with low pitches to the bottom and high pitches to the top. For a highly skilled cello player, this should have an impact on a grounded SPARC effect regardless of instrumental context and musical expertise (cf. Rusconi et al., 2006). Thus, one could hypothesize on the basis of embodiedness of GES that SPARC effects of highly skilled cello players would be reduced or even vanish. Why would this be the case? The grounded reference frame of cello players, like for others, is that high pitch is associated with upper locations and low pitch with lower locations in vertical space. However, the learned reference frame of cello players is that high pitch is associated with lower locations and low pitch with upper locations in vertical space, consistent with their long-term motor and perceptual experience on the cello. Accordingly, conflicting response codes of both reference frames might cause the SPARC effect to be reduced or to vanish, but presumably not to be reversed (cf. Wood, Nuerk, & Willmes, 2006, for an analog argument for the disappearance of SNARC for crossed hands).

However, it is unclear if the specificity of this experience would generalize to SPARC, in the sense that the particular spatial representation of tones of the cello might become a characteristic of cello players, for example, when listening to music. Rather, it might only appear in situations in which a cello tone is actually played. Thus, in an instrumental context evoked by showing the picture of a piano together with playing piano tones, the learned reference frame of cello players would not become active. This would result in a SPARC effect similar to that shown by non-musicians. In contrast, a reversed SPARC context created by showing the picture of a cello together with playing cello tones should activate the learned reference frame of cello players, causing the SPARC effect to be reduced or to vanish, because of the reversed vertical arrangement of cello tones. Such an effect would also be situated, because it depends on the specific experimental situation, namely the particular characteristics of the employed instrumental context.

With that experimental setup we were able to tackle groundedness, embodiedness and situatedness in SPARC. Accordingly, we formulated three hypotheses. First, if the SPARC effect is grounded, we should observe a standard vertical SPARC effect for non-musicians and musicians alike. This groundedness-hypothesis is predicted by GES due to the universality of representations grounded on universal physical properties or laws. The second hypothesis further narrows the first, namely if SPARC is embodied then a SPARC effect should be reduced or disappear in professional cello players regardless of instrumental context. This is due to their long-term learning experience of reversed SPARC associations (embodiedness-hypothesis). The third situatedness-hypothesis further concentrates on cello players, assuming if SPARC is situated then cello players should show a standard SPARC effect in a piano context (which would also support the groundedness-hypothesis for cello players) but none or a reduced SPARC effect in a cello context.

## Experiment 1

In this experiment we tested the paradigm with the intention to replicate the standard SPARC effect as in Rusconi et al. (2006) with a group of non-musicians. Experiment 1 set the baseline for the subsequent investigation with cello players.

### Participants

The sample consisted of 20 German students (16 female; 2 left-handed; the sample size was analog to the limited availability of cello players in Experiment 2) with a mean age of 24.85 years (SD 3.84 years). They had normal or corrected vision. All participants were naïve with regard to the research question and gave written consent to attend to the experiment. They had no musical expertise. As expense allowance, participants were paid 10 EUR.

### Material

**Fig. 1:**
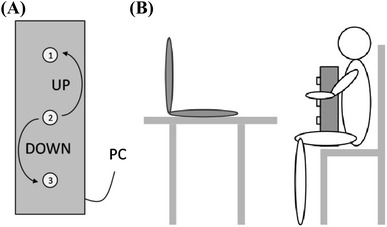
**a** Schema of the keyboard of vertically arranged keys used in the experiment. **b** Schema of the posture of the participant

The experiment ran in MATLAB on an Apple MacBook Pro. Subjects sat at a distance of 60 cm in front of it. Five tones at an interval of two semitone steps were used. The reference tone was ‘a2’ (110.0 Hz). The tones ‘g2’ (98.0 Hz) and ‘f2’ (87.31 Hz) served as primes lower than the reference, while ‘h2’ (123.47 Hz) and ‘cis3’ (138.59 Hz) served as higher primes, all recorded from a piano and from a cello. Tones were matched in length (1 s.) and volume both between and within the instrument-category. Cello tones were shortened at the end due to longer fading. Two other cello players affirmed that the shortened tones did not sound artificial or distorted, but highly suitable. High quality headphones (Sennheiser HD 201) were used to present the stimuli. Responses were recorded with an external PC-keyboard connected via USB to the MacBook. This keyboard was adapted with a locally constructed overlay (see Fig. 1a) consisting of three equidistant vertically arranged buttons. To set the instrumental context in each trial, pictures of a piano or a cello were presented at the center of the monitor with a size of 70 × 70 pixels.

### Procedure and design

Participants were asked to perform a tone discrimination task in which two succeeding tones were presented with the sound of either a piano or a cello. They held the keyboard in front of them and fixated it with their knees and right hand (Fig. 1b). The buttons of the keyboard were not visible to participants due to the setup. Moreover, subjects were asked to respond with their left hand, since cello players grasp the strings with their left hand. The first tone served as a reference, the second tone as the target. The task was to judge the pitch height of the second tone compared to the reference. Participants were asked to respond on the vertically arranged keyboard in two different ways. In the congruent condition, they had to press the higher (lower) button to indicate that the target tone was higher (lower) than the reference tone. In the incongruent condition, the keyboard mapping was reversed, i.e., pressing the higher (lower) button indicates that the target tone was lower (higher). The experiment was divided into two blocks for the cello and two for the piano with four preceding practice trials each. The order was counterbalanced across participants. Each trial was initiated by pressing and holding down the middle button on the keyboard. After that, the visual prime setting in the instrumental context was displayed. With its onset, the reference tone appeared for 2000 ms followed by a randomly chosen target tone. The response was given by releasing the middle button and pressing either the upper or lower button, depending on the response mapping, as fast as possible after the target tone appeared. The reaction time was measured as the time between the onset of the target tone and the release of the middle button. After 2000 ms without response the trial was canceled. In either case, participants had to start the next trial by pressing the middle button.

Each target tone was presented 20 times. Thus, for each participant 80 randomly presented trials per one of the four counterbalanced blocks were obtained, resulting in a total number of 320 trials (160 trials for piano, 160 trials for cello). The study was a full factorial 2 × 2 × 2 within-subject design with the factors “pitch height” (lower or higher compared to reference tone), “response direction” (up vs. down) and “instrumental context” (cello vs. piano).

### Data preparation

All analyses were conducted with R (R Core Team, 2016). Data are available under figshare data repository (Lachmair, 2016a). Three participants were excluded from further analyses due to an error rate above 20%. Another participant was excluded due to a mean reaction time larger than four standard deviations of the group mean. Reaction times less than or equal to 200 ms were excluded from the analysis, as were error trials.^1^ This reduced the data by 3.7%. Furthermore, reaction times deviating by more than three times the participant’s individual standard deviation were filtered out (5.33%). This was applied consecutively to the data of each participant (5 times in average, min_times_ = 2, max_times_ = 8) until there were no more outliers according to the updated standard deviation.

This procedure of correcting for outliers has been applied in many previous papers (e.g., Cipora & Nuerk, 2013). The statistical rationale is that when applying a correction per participant and condition, the estimates of means and variances are only suboptimal estimations of the true underlying mean and the true underlying variance, due to the rather small *N* in each condition, as in the present study. What is worse, however, is that outliers are not part of the distribution underlying the sample mean and the sample variance. They are different random variables (which is the reason we wish to exclude them). Thus, the important point is that exclusion of outliers relies on the computation of a sample mean and a sample variance, which is calculated with these outliers included. For small *N*, even few outliers greatly increase the mean and the variance. This mean and this variance will then largely deviate from the true mean and variance and may in fact include outliers that would be excluded if the true mean and variance were known. This is the reason for the repetitive exclusions, because the sample mean and the sample variance are likely much better estimators of true mean and true variance when outliers are excluded. The search for other outliers should be done with these better estimators and not with the first calculations, which included all outliers. Nevertheless, there is always the danger of eliminating true effects, when the mean is calculated over all conditions. This is more of a problem when the effect sizes are larger, which in typical psychological studies is not the case. This can be made clear with the following example, where a middle effect size (according to Cohen) of 0.5 and, for simplicity, equal standard deviations of the conditions are assumed. If values are cut off larger than three standard deviations (3 SD), the cumulative standard normal distribution value is 0.99865. That means, we wrongly cut off 0.135% of the values of the real underlying distribution. With the effect size of 0.5, values outside 2.75 SD were cut off in the slower condition, but outside 3.25 SD in the faster condition. The cumulative distribution values are 0.99702 and 0.99942, which means that 0.398% were cut out in one case and 0.058% in the other case of the true underlying trials. This makes a difference of 0.340%, which is approximately every 300th trial. Note that the N per condition in the present study is 20. Compared to the negative impact that missing outliers can have on the data, we believe this is negligible. Therefore, outlier correction over the full data set of an individual produces more stable results, because eliminating an extra 0.3% of the true underlying distribution is preferable to having missed outliers.^2^

### Results and discussion

**Fig. 2:**
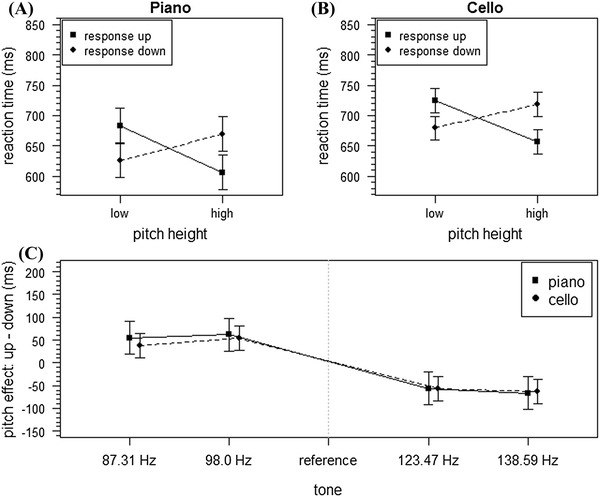
Results of Experiment 1. Mean reaction times (ms) of Correct Responses for **a** piano and **b** cello as a function of pitch height and response direction. Pitch-effect as a function of instrumental context and tone (**c**). *Error bars* represent the 95% confidence interval for within-subject designs (Masson & Loftus, 2003)

The data are illustrated in Fig. 2. With the remaining data an ANOVA was conducted with the categorical factors instrumental context, response direction and pitch height. First, the results showed a main effect for instrumental context [*F*(1, 15) = 11.02, *p* = 0.005, $\eta_{\text{p}}^{2}$ = 0.42] with faster reaction times for piano (647 ms) than for cello (695 ms). Moreover, the main effect for pitch height was significant [*F*(1, 15) = 6.93, *p* = 0.02, $\eta_{\text{p}}^{2}$ = 0.32] with faster reaction times for high pitches (663 ms) compared to low pitches (678 ms). There was no main effect of response direction [*F*(1, 15) = 1.57, *p* = 0.23]. The two-way interactions between instrumental context and pitch height as well as instrumental context and response direction and the three-way interaction between instrumental context, response direction and pitch height were not significant (*F*s < 1, *p*s > 0.36). However, pitch height interacted significantly with response direction [*F*(1, 15) = 21.02, *p* < 0.001, $\eta_{\text{p}}^{2}$ = 0.58], showing faster reaction times in congruent (high pitch—top response: 632 ms, low pitch-bottom response: 653 ms) compared to incongruent conditions (low pitch—top response: 704 ms, high pitch—bottom response: 694 ms; see Fig. 2). Keeping in mind controversies regarding the confirmation of a null hypothesis using traditional statistical inference, we employed a Bayesian method. The method described in detail by Masson (2011) enables calculating graded evidence for a null hypothesis (i.e., no difference between groups) and alternative hypothesis (i.e., difference between groups). In the analysis, the sum of squares and number of observations from ordinal analysis of variance (ANOVA) are used to calculate Bayesian factors, which then can be used to calculate posterior probabilities. Based on our data, the posterior probability of null hypothesis for the non significant three-way interaction was 0.77 (alternative hypothesis 0.23). Applying the criteria suggested by Masson (2011; see also Raftery, 1995), this is positive evidence for the null hypothesis, assuming no difference between instrumental contexts in terms of pitch height and response direction for non-cellists.

### Regression analysis of pitch

To gain further insight to the effect of pitch, the analysis proposed by Fias (1996) was conducted. Therefore, averaged reaction times (RT) of each subject per tone, response direction and instrumental context were calculated. Afterwards, the differences of these means (dRTs) were calculated, i.e., the mean RT of upward responses minus the mean RT of downward responses. This difference by definition was positive when upward responses were slower than downward responses and negative when upward response were faster than downward responses. The dRTs were then regressed on pitch height (coding: ‘f2’ = −2, ‘g2’ = −1, ‘h2’ = 1 and ‘cis3’ = 2) using linear regression for each participant separately. The regression slope can be interpreted as a measure of the SPARC effect: The more negative the value, the stronger the SPARC effect. The mean slope was −33.65 (SD 32.48). The presence of a SPARC effect was examined by testing slopes against 0 using a one-sample *t* test. Levene’s test (*F* = 0.24, *p* = 0.63) showed no indication of unequal variances between cello and piano for SPARC slopes; therefore an unadjusted *t* test was used. The SPARC effect was significant (*t*(31) = −5.86, *p* < 0.001). Slopes for the Piano context were not significantly different from those for the cello context [*t*(15) = −0.76, *p* = 0.46; *M* = −36.25, SD = 37.41, and *M* = −31.06, SD = 27.69, respectively].

## Experiment 2

The first experiment showed a SPARC effect on a vertical spatial axis for non-musicians which was unaffected by instrumental context. This suggests a grounded SPARC for non-musicians, in the absence of alternative embodied music-space mappings. The second experiment examined whether musical expertise would reveal additional characteristics moderating or even overwriting the groundedness direction of the SPARC. Therefore, the second experiment was conducted by employing the same paradigm as in Experiment 1, but this time with highly skilled cello players. As outlined above, learning experience of the cello may reverse basic grounded music-space associations: high tones are played downwards and low tones upwards on the cello.

### Participants

The sample consisted of 20 cello players (8 female; *M*_age_ = 21.10 years, SD = 4.34) with a high level of musical expertise (*M*_expertise_ = 12.12 years, SD = 4.25; with at least 2 h exercise a week). All participants were right-handed, naïve with regard to the research question and gave written consent to attend to the experiment. As expense allowance, participants were paid 10 EUR.

### Material

The same material was employed as in Experiment 1.

### Procedure and design

The same procedure and design was employed as in Experiment 1.

### Data preparation

For the analysis, the data of all participants were included. Data are available under figshare (Lachmair, 2016b). One percent of trials were excluded due to false responses.^3^ Responses faster than 200 ms were also excluded from further analysis (ca. 1%). The trimming procedure of the data as in Experiment 1 (3.8 times in average for each participant, min_times_ = 1, max_times_ = 7) reduced the data by 2.88%.

### Results and discussion

**Fig. 3:**
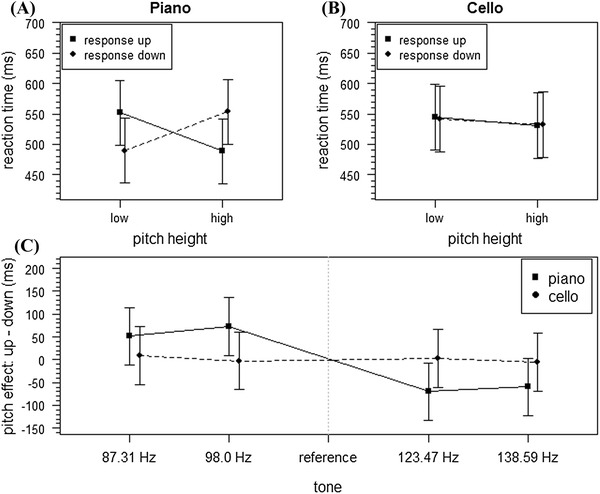
Results of Experiment 2. Mean reaction times (ms) of correct responses for **a** piano and **b** cello as a function of pitch height and response direction. Pitch effect as a function of instrument and tone (**c**). *Error bars* represent the 95% confidence interval for within-subject designs (Masson & Loftus, 2003)

Data are illustrated in Fig. 3. With the remaining data, reaction times were analyzed with an ANOVA that was conducted with the categorical factors instrumental context, response direction and pitch height. First, the results showed a marginal significant main effect for the factor pitch height [*F*(1, 19) = 3.88, *p* = 0.06, $\eta_{\text{p}}^{2}$  = 0.17]. Moreover, the interaction between

pitch height and instrumental context was significant (*F*(1, 19) = 4.95, *p* = 0.04, $\eta_{\text{p}}^{2}$ = 0.21] with equal reaction times in the piano condition (low pitch 521 ms, high pitch 521 ms) and different reaction times in the cello condition (low pitch 543 ms, high pitch 531). All other factors and two-way interactions were not significant, particularly the two-way interaction between pitch height and response direction (*F*s < 1.4, *p*s > 0.23). Bayesian analysis for the latter showed a posterior probability for null hypothesis of 0.68. Applying the criteria suggested by Masson (2011; see also Raftery, 1995), this is weak evidence for the null hypothesis, assuming no interaction between pitch height and response direction for cello player regardless of instrumental context. Moreover, interestingly, the three-way interaction between pitch height, response direction and instrumental context was significant [*F*(1, 19) = 4.58, *p* = 0.046, $\eta_{\text{p}}^{2}$ = 0.19]. A closer investigation of this three-way interaction revealed that this was due to a significant two-way interaction between pitch height and response direction in the piano condition [*F*(1, 19) = 4.40, *p* = 0.049, $\eta_{\text{p}}^{2}$ = 0.19] with faster responses in congruent (high pitch—top response: 489 ms, low pitch-bottom response: 490 ms) compared to incongruent conditions (low pitch—top response: 552 ms, high pitch—bottom response: 553 ms; see Fig. 3). However, this two-way interaction disappeared in the cello condition [*F*(1, 19) = 0.01, *p* = 0.94]. Based on these data and using Bayesian analysis as conducted in Experiment 1, the posterior probability of the null hypothesis of these two-way interactions was 0.36 for piano condition and 0.82 for cello condition. Applying the criteria suggested by Masson (2011; see also Raftery, 1995), this is positive evidence for an interaction between pitch height and response direction in the piano condition and strong evidence for a null interaction in the cello condition.

### Regression analysis of pitch

We conducted the same regression analysis for pitch as in Experiment 1. The mean slope was −19.31 (SD 82.69). The overall SPARC effect was not significant [*t*(39) = −1.48, *p* = 0.15]. However, as predicted in our third hypothesis, slopes for the Piano context were significant and significantly larger than for the cello context [*t*(19) = −2.01, *p* < 0.05, one-sided; *M*_piano_ = −36.34, SD_piano_ = 80.73 and *M*_cello_ = −2.28, SD_cello_ = 83.10, respectively; see Fig. 3]. Levene’s test (*F* = 0.00, *p* = 0.99) showed again no indication of unequal variances for SPARC slopes.

## General discussion

In the present study we tested highly skilled cello players against non-musicians with regard to their space-pitch associated response codes, so-called SPARC. Although several studies have already examined SPARC for various musicians, the present study makes a significant contribution regarding the different experiential sources of SPARC. This will be discussed in detail in the following.

As previously described, several studies have shown that musical expertise can affect SPARC due to a strong perception-motor coupling which is based on the spatial arrangement of pitch on a music instrument (e.g., piano or guitar) and intensive and long-lasting training experience with that instrument (e.g., Repp & Knoblich, 2007, 2009; Taylor & Witt, 2015; Drost et al., 2005). Typically, these SPARC effects have a horizontal spatial alignment, reflecting the space-pitch alignment of the instrument. It is notable (1) that these alignments can be from left to right as well as from right to left depending on the pitch alignment of the respective instrument and (2) that the apparent strength of these effects does not depend on these alignments per se. This speaks for a rather high flexibility of SPARC depending on the pitch alignment of the instrument (cf. Stewart et al., 2013a, b). In addition, there is also evidence of SPARC effects for people without musical expertise. For example, this has been shown in a study by Rusconi et al. (2006) comparing piano players with non-musicians. Interestingly, whereas only the musicians showed a horizontal SPARC effect, this study showed that both musicians and non-musicians showed a vertical SPARC effect with low pitch associated to lower space and high pitch to upper space (Rusconi et al., 2006). But considering that non-musicians do not have profound experiences stemming from practicing with a certain music instrument, the conclusion is that horizontal and vertical SPARC effects must have different sensorimotor sources.

A brief discursion, which does not immediately appear related to research on SPARC, may help to clarify why it is reasonable and maybe important to differentiate spatial alignments as experiential sources of sensorimotor bindings. From astronauts aboard the International Space Station (ISS) it is known that losing gravity during microgravity exposure in orbit challenges human spatial cognition, as manifested by spatial disorientation, space motion sickness, and cognitive deficits. The reason is presumably that the human vestibular system has developed in the context of the gravitational force of the earth and uses it as a ubiquitous reference for the vertical space which is no longer available (see reviews by Clément & Reschke, 2008; Lackner & DiZio, 2000; Casler & Cook, 1999). Interestingly, the reported issues are related exclusively to vertical space and although an adaptation can succeed, it can take up to 3 months (e.g., Clément & Reschke, 2008). Compared to this, it seems that adapting to the reverse horizontal alignment of numbers on a keyboard, for example, would succeed with far less effort.

Interestingly, existing theories like the Theory of Event Coding (TEC; Hommel, Musseler, Aschersleben, & Prinz, 2001) do not account for these characteristics. For example, a horizontal SPARC effect based on practicing with a music instrument would be seen in the same way as a vertical SPARC effect, which is, however, grounded on fundamental physical laws. The GES-framework takes these differences into account. It considers grounded, embodied and situated experiences as different sources which are manifested in vertical or horizontal space (e.g., Fischer & Brugger, 2011; Myachykov et al., 2014).

In the present study, we tested GES by comparing SPARC of non-musicians with SPARC of cello players. GES predicts that according to the groundedness of pitch, both groups would show a vertical SPARC with low pitch to the bottom and high pitch to the top. Further, due to the learning experiences with the cello and its reversed space-pitch alignment, GES predicts also an embodied influence on the vertical SPARC for cello players. However, it can be assumed that due to the generality of the vertical SPARC this influence is not strong enough to reverse the SPARC for cello players. The expectation was rather that the vertical SPARC would be reduced or even disappears, but would not be reversed. Moreover, the instrumental context should influence the vertical SPARC for cello players, which would reflect the situatedness of SPARC (e.g., Drost et al., 2007). Accordingly, it was expected that the standard vertical SPARC would be influenced in the context of cello-timbre but not in the context of piano-timbre.

In Experiment 1, for non-musicians a strong SPARC effect was found regardless of instrumental context. Due to the lack of learning and practicing experiences with the employed instruments no embodiedness or situatedness of SPARC was expected nor has been found. Thus, this SPARC effect can be considered grounded. As suggested by Rusconi and colleagues this groundedness may arise from a general sense of magnitude. It is built upon an omnipresent spatial reference frame based on general physical constraints, which is automatically activated when processing pitch, associating low pitch with the bottom and high pitch with the top of vertical space (cf. Wood et al., 2006). The latter is supported by the study of Parise et al. (2014), suggesting that higher tones more likely stem from sources in higher locations and lower tones from sources in lower locations according to natural scene statistics (Parise et al., 2014). Note, in the present study the spatial dimension of the auditory stimuli was task-relevant. With regard to automaticity of SPARC this could be problematic (cf. Firestone & Scholl, 2014). However, first, the study refers to and was built upon the findings of Rusconi et al. (2006), already showing a vertical SPARC for musicians and non-musicians alike. They have shown this effect with a paradigm where the stimulus dimension was task-relevant and also with a paradigm where the stimulus dimension was task-irrelevant. So we can assume that a vertical SPARC exists and that it is highly automatic. Second, the intention of the present study was to find interactions for the groups of cello player and non-musicians in the two different instrumental contexts. Thus, we believe that task relevance of stimulus dimension is negligible for this study.

The results of Experiment 2 show additional characteristics of embodiedness and situatedness for the SPARC of cello players. This was confirmed by the significant three-way interaction between pitch height, response direction and instrumental context. Examining this interaction revealed a standard SPARC effect in the piano context, but no SPARC effect in the cello context. First, this confirmed the situatedness-hypothesis for cello players, showing that the instrumental context in the experiment did influence the SPARC effect of this group. However, due to the experimental setup implying images of piano and cello statically paired with the related instrumental timbre of cello and piano, it is not clear if the situatedness in the present study relies on the image, the timbre or both. The intention was to maximize the impact of the situatedness by manipulating sound and picture together without the use of a real graspable instrument. On the basis of the literature, we can, however, suggest that sound could be (partially) responsible as it was leading to action-interference effect in musicians, which were, however, not linked to space. Because spatial associations with other metrics can be linked to visual attentional preferences (e.g., Fischer et al., 2003), we may also assume that the visual picture may play a role. Our hypothesis, therefore, would be that both modalities and possibly their interaction play a role. However, this needs to be disentangled in future studies.

Second, the embodiedness-hypothesis is also corroborated by this null interaction for cello tones and the weaker SPARC effect for piano tones, especially when both are compared to the SPARC effect of non-musicians. This null interaction can be explained in analogy to the reasoning of Wood et al. (2006) about different conflicting reference frames in the SNARC-effect. In their work the authors found no SNARC-effect in a parity judgment task when responding hands were crossed (right hand on the left side and left hand on the right side). They argued that under this condition two different reference frames were activated simultaneously, one referring to (external) space (small numbers are associated to the left and large numbers to the right) and the other referring to hand (right hand is associated to large and left hand to small numbers). This leads to conflicting response codes, one hand-based, one space-based, pointing into different directions and thereby eliminating the SNARC-effect. An analogous mechanism might describe the obtained results for cello players. Here we look at a grounded and an embodied reference frame, the latter based on long lasting learning experiences with the cello. The grounded reference frame associates low pitches with the bottom and high pitches with the top corresponding to our external world. The embodied reference frame for cello player associates vice versa, high pitches with the bottom and low pitches with the top corresponding to their long-term cello playing experience. These conflicting spatial associations could have caused the elimination of the SPARC effect (cf. Wood et al., 2006). In contrast, TEC for example would either predict the grounded SPARC effects or a reversed SPARC effect for cello players in the cello condition, ignoring the interplay between them. Thus, it is important to note that the elimination of SPARC was the most pronounced in situations were the cello tones were played to activate the cello experience of cello players.

Therefore, we have an interaction of embodiment and situatedness in our study. The modulation of the SPARC effect for cello players is not purely embodied, because then we should have also found it in other situations, namely when piano tones were played. Nevertheless, the SPARC effect is reduced compared to non-musicians. On the other hand, the effect of cello tones is not purely situated. Cello tones themselves do not change the SPARC effect in general. They only do so when they are connected to long-term cello playing experience, but are irrelevant for non-musicians with no cello experience.

The present study is different from the study by Drost et al. (2007) in several respects. Drost et al. showed integrated action effects, i.e., that playing a tone or a chord on piano and guitar can be subject to interference when a different tone or chord is auditorily represented. The important theoretical difference is that in the present study, we examined directional music-space associations and their mental representations, while Drost et al. (2007) examined action effects themselves. This can be seen in their experimental setup: the participants played the tone either on a piano-like keyboard or grasped the chord on a guitar. In our study, no action on an instrument was required, nor was an instrument even present. All that were present were an auditory tone and an image of the instrument to facilitate the mental representation induced by that instrument. Importantly, the mental representation induced was just related to a vertical position (up or down) and not to a specific chord like “A major” or “A minor”. In that sense, the music–space associations we examined in the present study were much less concrete and seemingly more abstract than the particular instrumental actions of Drost et al. (2007). Having said this, we believe that our data are complementary to the findings of action-effect studies like for example in Drost et al. (2007). The data of this study can also be explained in the GES-framework, showing that situated elements (piano and guitar tones) can interfere with specific action effects. They do not show grounded effects, which are difficult to show for concrete action effects anyway. They also do not show embodied effects as they did not compare experienced musicians with non-musicians. With regard to situated effects for musicians, this can serve as an additional motivation to expect such effects not only for action integration but for mental representations in general.

To sum up, the present study sheds some light on the different experiential sources of SPARC, namely groundedness, embodiedness, and situatedness showing strong groundedness of SPARC, fundamentally. However, high musical expertise and instrumental context can selectively influence SPARC: activating long-term learning experiences from playing the cello within a cello context is capable of extinguishing the effect. This is presumably due to a simultaneous activation of two conflicting reference frames with different strengths: the grounded reference frame of standard SPARC and the embodied reference frame of reversed SPARC. This demonstrates again the great embodied and situated flexibility and temporary nature of spatial representations used to represent cognitive metrics; beyond fundamental representational aspects due to physical properties and laws this flexibility and fluid temporal nature, known from representing number-space mappings, language-space mappings and finger-number mappings, also extends to music. Therefore, we suggest that the three experiential sources of groundedness, embodiedness and situatedness constitute a general characteristic of mapping cognitive representations to space.

## Footnotes

1 An error analysis showed no significant interactions (*F*s < 2.28, *p*s > 0.15).

2 An alternative analysis with outlier correction per participant and condition did not affect substantially the results.

3 An error analysis showed no significant interactions (*F*s < 2.21, *p*s > 0.15).
